# Supplementary material for: A Peer-Based Educational Intervention Effects on SARS-CoV-2 Knowledge and Attitudes among Polish High-School Students
Source: Int J Environ Res Public Health. 2021 Nov 20;18(22):12183. doi: 10.3390/ijerph182212183 (PMC8623814; doi:10.3390/ijerph182212183)
Supplement: Supplementary file 1 [file ijerph-18-12183-s001.zip › ijerph-1416001-supplementary.pdf]

Table S1. Frequency of Correct Answers in General Knowledge About SARS-CoV-2 and COVID-19 Pre- and Postintervention by Gender, division and school location; Final year high school Students, Poland, 2020 (N = 518) .

Statement	Correct Answer	Category	Total n	Correct Answers				p Value for Change (*Negative)
				Preintervention		Postintervention		
				n	%	n	%	
The main clinical symptoms of COVID-19 are fever, fatigue, muscle ache and dry cough	True	Total	518	485	94%	489	94%	0.52
		Males	176	158	90%	166	94%	0.046
		Females	342	327	96%	323	94%	0.39
		Life science	376	357	95%	358	95%	0.84
		Other	142	128	90%	131	92%	0.41
		<250 000	376	352	94%	356	95%	0.43
		≥250 000	142	133	94%	133	94%	<0.0001
Unlike the common cold, stuffy or runny nose and sneezing are less common in people infected with SARS-Cov-2	True	Total	518	261	50%	363	70%	<0.0001
		Males	176	78	44%	124	70%	<0.0001
		Females	342	183	54%	239	70%	<0.0001
		Life science	376	203	54%	272	72%	<0.0001
		Other	142	58	41%	91	64%	<0.0001
		<250 000	376	182	48%	262	70%	<0.0001
		≥250 000	142	79	56%	101	71%	0.0002
Currently there is no effective cure for COVID-19	True	Total	518	432	83%	458	88%	0.005
		Males	176	140	80%	153	87%	0.03
		Females	342	292	85%	305	89%	0.07
		Life science	376	314	84%	336	89%	0.005
		Other	142	118	83%	122	86%	0.41
		<250 000	376	317	84%	335	89%	0.34
		≥250 000	142	115	81%	123	87%	0.12
Elderly, obese and chronically ill people are at greater risk of severe COVID-19	True	Total	518	493	95%	503	97%	0.06
		Males	176	162	92%	169	96%	0.07
		Females	342	331	97%	334	98%	0.41
		Life science	376	364	97%	366	97%	0.64
		Other	142	129	91%	137	96%	0.01
		<250 000	376	353	94%	364	97%	0.02
		≥250 000	142	140	99%	139	98%	0.65
The origin of SARS-CoV-2 is natural transmission from animals to humans, also called zoonotic transmission	True	Total	518	232	45%	441	85%	<0.0001
		Males	176	81	46%	147	84%	<0.0001
		Females	342	151	44%	294	86%	<0.0001
		Life science	376	178	47%	323	86%	<0.0001
		Other	142	54	38%	118	83%	<0.0001
		<250 000	376	155	41%	309	82%	<0.0001
		≥250 000	142	77	54%	132	93%	<0.0001
People infected with SARS-Cov-2 cannot infect others unless they are febrile	False	Total	518	451	87%	458	88%	0.38
		Males	176	144	82%	145	82%	0.84
		Females	342	307	90%	313	92%	0.33
		Life science	376	343	91%	344	91%	0.87
		Other	142	108	76%	114	80%	0.22
		<250 000	376	318	85%	328	87%	0.14
		≥250 000	142	133	94%	130	92%	0.47
SARS-Cov-2 is spread by droplets released from the respiratory tract of infected people	True	Total	518	474	92%	505	97%	<0.0001
		Males	176	149	85%	167	95%	0.0001
		Females	342	325	95%	338	99%	0.002
		Life science	376	356	95%	368	98%	0.005
		Other	142	118	83%	137	96%	<0.0001
		<250 000	376	335	89%	364	97%	<0.0001
		≥250 000	142	139	98%	141	99%	0.32
Isolating and treating people infected with SARS-Cov-2 are effective ways to reduce the viral spread	True	Total	518	456	88%	494	95%	<0.0001
		Males	176	146	83%	164	93%	0.0004
		Females	342	310	91%	330	96%	0.0006
		Life science	376	339	90%	364	97%	<0.0001
		Other	142	117	82%	130	92%	0.005
		<250 000	376	322	86%	355	94%	<0.0001
		≥250 000	142	134	94%	139	98%	0.06

Convalescents from SARS-Cov-2 infection get lifelong immunity	False	Total	518	300	58%	424	82%	<0.0001
		Males	176	92	52%	138	78%	<0.0001
		Females	342	208	61%	286	84%	<0.0001
		Life science	376	238	63%	326	87%	<0.0001
		Other	142	62	44%	98	69%	<0.0001
		<250 000	376	202	54%	297	79%	<0.0001
		≥250 000	142	98	69%	127	89%	<0.0001
Currently, the reported number of SARS-CoV-2 infections worldwide is between 20-50 million	True	Total	518	150	29%	360	69%	<0.0001
		Males	176	58	33%	136	77%	<0.0001
		Females	342	92	27%	224	65%	<0.0001
		Life science	376	115	31%	270	72%	<0.0001
		Other	142	35	25%	90	63%	<0.0001
		<250 000	376	95	25%	242	64%	<0.0001
		≥250 000	142	55	39%	118	83%	<0.0001
Currently, the reported number of SARS-CoV-2 infections in Poland does not exceed 1000	True	Total	518	156	30%	309	60%	<0.0001
		Males	176	70	40%	116	66%	<0.0001
		Females	342	86	25%	193	56%	<0.0001
		Life science	376	115	31%	240	64%	<0.0001
		Other	142	41	29%	69	49%	<0.0001
		<250 000	376	111	30%	208	55%	<0.0001
		≥250 000	142	45	32%	101	71%	<0.0001
SARS-CoV-2 incubation period	2-14 days	Total	518	449	87%	485	94%	<0.0001
		Males	176	141	80%	159	90%	0.0002
		Females	342	308	90%	326	95%	0.003
		Life science	376	336	89%	361	96%	<0.0001
		Other	142	113	80%	124	87%	0.01
		<250 000	376	320	85%	346	92%	0.0002
		≥250 000	142	129	91%	139	98%	0.004
Currently, remdesivir, convalescent plasma and supportive therapy are used to treat COVID-19	True	Total	518	42	8%	218	42%	<0.0001
		Males	176	15	9%	77	44%	<0.0001
		Females	342	27	8%	141	41%	<0.0001
		Life science	376	35	9%	174	46%	<0.0001
		Other	142	7	5%	44	31%	<0.0001
		<250 000	376	26	7%	142	38%	<0.0001
		≥250 000	142	16	11%	76	54%	<0.0001
COVID-19 protective measures are as follows: wearing a mask, social distancing, frequent hand-washing, the avoidance of touching eyes, nose & mouth	True	Total	518	347	67%	415	80%	<0.0001
		Males	176	104	59%	130	74%	<0.0001
		Females	342	243	71%	285	83%	<0.0001
		Life science	376	268	71%	312	83%	<0.0001
		Other	142	79	56%	103	73%	<0.0001
		<250 000	376	246	65%	302	80%	<0.0001
		≥250 000	142	101	71%	113	80%	0.03
